# Attitude confidence and source credibility in information foraging with social tags

**DOI:** 10.1371/journal.pone.0210423

**Published:** 2019-01-15

**Authors:** Stefan Schweiger, Ulrike Cress

**Affiliations:** 1 Knowledge Construction Lab, Leibniz-Institut fuer Wissensmedien, Tuebingen, Germany; 2 University of Tuebingen, Tuebingen, Germany; Coventry University, UNITED KINGDOM

## Abstract

There is growing concern that online information searchers are overconfident and therefore largely search for information which reinforces their prior attitudes, blinded by confirmation bias. This study tests if this effect can be reduced in content aggregation platforms, when social tag clouds show popular topics among experts. We manipulated (1) confidence in prior attitudes, and (2) the credibility of the expert community that tagged the content. We found that both factors influence navigation in different ways. First, attitude confidence moderated the influence of prior attitudes when choosing how much attitude-consistent content in blog posts to read. When attitude confidence was high, prior attitudes were positively associated with selection of blog posts, when low, not positively associated. After navigation, when confidence was high, the content of attitude-consistent blog posts was more favourably evaluated, whereas when confidence was low, attitude inconsistent blog posts were more favourably evaluated. Second, source credibility moderated the influence of prior attitudes on tag selection. When source credibility was low, prior attitudes did guide tag selection, when high, they did not. With low source credibility, people selected more attitude-consistent content. The findings advance social tagging theories by showing that not only semantic associations, but also attitudes play a role when people select and process tags and related content. The findings also show that credibility and confidence have a different impact on different stages of information selection and evaluation. Whereas credibility is more important when switching among pages, attitude confidence is more important when reading and evaluating the content of one page.

## Introduction

In online environments, it has been suggested that we often find ourselves in a filter bubble or echo chamber, where we only receive and attend to information that is consistent with our views and prior attitudes (e.g. [1–10]). This is called confirmation bias [1,2], and its robustness has been extensively documented by literature (see [1,2] for an overview and [3] for a meta-analysis). Confirmation bias is particularly prevalent and pertinent in online content platforms [4–7,10], and it fundamentally shapes our search for, and evaluation of information [1,11]. Confirmation bias might possibly set us on a particularly harmful track, for example, when we search for health-related information online [10].

Online search is so important that people still prefer to search online for an answer to a question even if they have an answer at hand [12]. This also suggests that searchers have less confidence in their own knowledge than in information they find online [12]. But people are also exceedingly confident about their knowledge in spite of actual limitations to that knowledge. Such confidence has been exhibited in a large range of domains [13]. These findings show the central role confidence plays in online information search. Thus, the question of the consequences of confidence on confirmation bias is a fundamental one. In the study presented here, we investigate whether confirmation bias increases when searchers are highly confident and perceive their own attitudes as highly valid.

Besides confidence, perceived validity of one’s own attitudes and perceived validity of the online community’s knowledge could also influence confirmation bias. However, this implies that searchers are equipped with the skill of recognizing whether a source is credible or not. There is mixed evidence from different online platforms whether people succeed in taking source credibility adequately into account. A recent review found in the context of health-related search that homophily drives credibility evaluations of user-generated content [14]. That is, blog posts and health forum entries are evaluated as more credible when authors of blog posts or forum entries have demographics similar to those of the searcher [14]. But in social media or content aggregation platforms, the creator of the content often differs from the person or community which collects and shares content. We test whether searchers evaluate credibility correctly in terms of expertise of the community. And we ask the question whether the perception that the community is highly credible makes the searcher more open to content in spite of their prior attitudes.

Finally, we explore how confidence and credibility shape confirmation bias in different stages of information search. To do this, we draw on the most influential theory of human information search, the information foraging theory [15], as well as the extended information scent model [16]. Information foraging theory distinguishes between the breadth and depth of navigation. There are two fundamental search processes: the *navigation among* information patches (e.g., different websites) and the *uptake of information within* one information patch (e.g., a single website). With respect to confirmation bias, this has far reaching implications: Individuals with higher attitude confidence may be prone to explore more attitude-consistent information patches, and dwell on attitude-consistent information within each patch. On the other hand, when searchers perceive the community that created and tagged the content as more credible and knowledgeable, they may be open to move among patches and read more content within single patches, independently of their prior attitudes. Interestingly, to our knowledge, no studies thus far have investigated confirmation bias with respect to both of these online search activities. In addition, we explore whether factors such as credibility or attitude confidence influence both information search activities.

In sum, our expectations are the same for both selection activities (between-patch and within-patch). As for the health-related context, we expect people to try to find the objectively best possible treatment, not a treatment that validates their self-concept [3]. So, when searchers doubt the objective correctness of their prior attitudes because of lowered attitude confidence, they should be more open to attitude-inconsistent information. When searchers hold their prior attitudes with high confidence, they should select more attitude-consistent information. On the other hand, when searchers doubt the objective correctness of the information provided by a tagging community where source credibility may be low, they should select less information in general, independently of prior attitudes. In the following section we will review literature relevant to these issues.

### Attitude confidence and confirmation bias

People are, in general, exceedingly confident in the correctness of their knowledge and attitudes a confidence which is often inconsistent with their actual abilities. This is a phenomenon found in a wide range of domains, such as academic, intellectual, vocational, athletic, or even medical domains (for a review see [13]; [17–20]). At the same time, people don’t trust the confidence in their own knowledge enough, instead preferring to search online for an answer to a question, even when they already know the answer [12]. Moreover, attitude confidence varies in different situations and domains. For example, when searching for health-related information, people may find themselves becoming uncertain when they must cope with diagnosis or a disease [21,22]. Having online information at hand even appears to increase overconfidence, as information searchers mistake access to information as having knowledge [23]. So, the question arises, how does heightened or lowered confidence diminish or mitigate confirmation bias?

Confidence, in general, has manifold consequences for selection and judgment of information. For example, people who are highly confident will select information in line with their prior attitudes [24]. On the other hand, a meta-analysis has shown that high confidence tends to decrease confirmation bias, since people don’t experience any threat to their own point of view (low defense motivation [3]). It should be noted, however, that the meta-analysis also presented a number of studies where high confidence did not decrease but instead increased confirmation bias [3].

We expect that high attitude confidence would increase confirmation bias. This is because when we manipulate the metacognitive aspect of attitude confidence, we assume that defense motivation would not increase [3], but that the individual’s estimation of the validity of their own prior attitudes would increase. When individuals view their prior attitudes to be valid, confirmation bias should increase. When they estimate their attitudes to be invalid, confirmation bias should decrease. This is in line with the accuracy motivation theory, which in the health-related context, implies that information searchers should be motivated to search for objectively correct, valid information.

To illustrate this, there is only one study that used the same manipulation of attitude confidence as we have used it (Study 3; [25]). So in this respect, the study presented here is a replication attempt. In the original study using manipulation of confidence, students first read a strong or weak version of a persuasive message in favor of comprehensive exams. In response to this, they were asked to provide their thoughts about the message. Then, for an alleged unrelated study, students were instructed to think back to situations in which they experienced doubt or confidence about their own thoughts. They were subsequently instructed to think back about the thoughts they had had in response to the persuasive message they had just experienced. Students who had recalled previous situations in which they were highly confident were more confident about their thoughts regarding the message than students who had recalled previous situations in which they were doubtful. The degree of confidence also had consequences for the degree of persuasion. Participants who were highly confident about their thoughts in response to the message were also more persuaded in line with their thoughts. This study showed that high metacognitive confidence led to more confirmation bias with respect to content evaluation. Moving beyond this result, we go further here to test if high metacognitive confidence will lead to more attitude consistent information selection.

To conclude, as a result of undergoing metacognitive confidence manipulation in a health-related context, searchers’ information selection and evaluation should change as a function of attitude confidence. Highly confident searchers should believe in the validity of their prior attitudes. In contrast, less confident searchers should lose confidence in the validity of their attitudes and thus should look for more attitude inconsistent information and evaluate attitude inconsistent information more favorably.

### Source credibility in online information search

Social tagging platforms and other Web 2.0 environments like social networks are often characterized by the absence of professional gatekeepers who critically filter and select high quality content [26]. Therefore, it lies in the hands of the information searcher to critically evaluate sources of information themselves. In the following section, we will review the findings regarding whether people succeed in considering source credibility adequately, both in the online health-related context, and in online content aggregation platforms, such as social tagging environments.

A recent meta-analysis has shown that in the health context, it makes a difference whether people search for information on general websites or on sites that deliver user-generated content [14]. For websites, expertise was the decisive factor in credibility evaluations. But for user-generated content, homophily was decisive in credibility evaluations, so that searchers perceived laypersons who created content to have more credibility when their demographics were similar [14].

Also in the health-related context, but using web search engines, only some people succeeded in considering source credibility [27]. Among laypersons who searched for two competing treatments of Bechterew’s disease, those participants who viewed the web as a highly (vs. less) reliable, accurate source of knowledge failed to verbally reflect on the credibility of the information source. Therefore, they visually inspected URLs for shorter periods, and they were less likely to select results at the bottom of the search engine results (thereby failing to consider differences between search results; [27]). In a follow-up study, those participants who viewed the web as a highly (vs. less) reliable source of knowledge, spent less time on pages with objective information, and were less likely to base their treatment recommendation and evaluation on objective pages [28]. In this case, a short intervention improved their source evaluation behavior.

With respect to the visual presentation of search results, a study found that when search results were presented in a gridlike interface, similar to tag clouds, source evaluation became more important than when the presentation was made in list format, where also the influence of list position decreased while participants selected more trustworthy search results [29]. So, there is evidence that people succeed in critically evaluating information sources [30,31], but this depended on personal characteristics as well as the visual characteristics of the navigation interface [27].

Regarding the source evaluation of health-related information in social tagging systems, we found a single study which investigated whether people select tags that indicate the credibility of the tag-related source [32]. When participants who had been diagnosed with diabetes browsed a tag cloud with information on their condition, only one-third used at least one credibility-related tag. When explicitly asked to search for highly credible content, about 90% used at least one credibility-related tag, and only 23% exclusively used credibility-related tags. So, even when explicitly asked to evaluate source credibility, participants only partially evaluated credibility. In contrast to this, the study presented here does not focus on tags indicating credibility, but on the credibility of the community that provides the tags.

It is our aim to assess whether searchers adequately consider the source credibility of the tagging community. We varied the expertise of the community providing tags and expected that high expertise would relate to a perception that the community was more credible as a source. Blog post authors and cited sources within blog posts were constant and tested in a prior study. In line with the accuracy motivation theory for confirmation bias [3], we expected that perception of a community as highly credible should increase openness to selecting tags and blog posts, and increase openness towards persuasive messages from blog posts.

### Information foraging in tagging environments

In its description of the search for information, the information foraging theory is based on an analogy to food-foraging strategies in behavioral ecology [15]. One of the basic claims is that cognitive systems aim to maximize gains of valuable information in relation to search cost, analogous to animals’ food search. The information searcher (predator) browses different web sites (different patches in the environment) by estimating which links lead to highly valuable information (high yield patches, with lots of high caloric prey).

There are two fundamental activities in information foraging: between-patch exploration, describing the breadth of information search, and within-patch exploitation describing the depth of search and consumption of information. A searcher will switch between patches if the next patch promises higher information value than the current patch. In contrast to between-patch exploration, within-patch exploitation is the activity of processing information within a website. To operationalize navigation between and within information sources, we use a tagging environment where searchers navigate between patches (separate sites) via social tags. The tags lead searchers to patches that list several blog post headlines on which they can click to read the full blog post.

**Fig 1:**
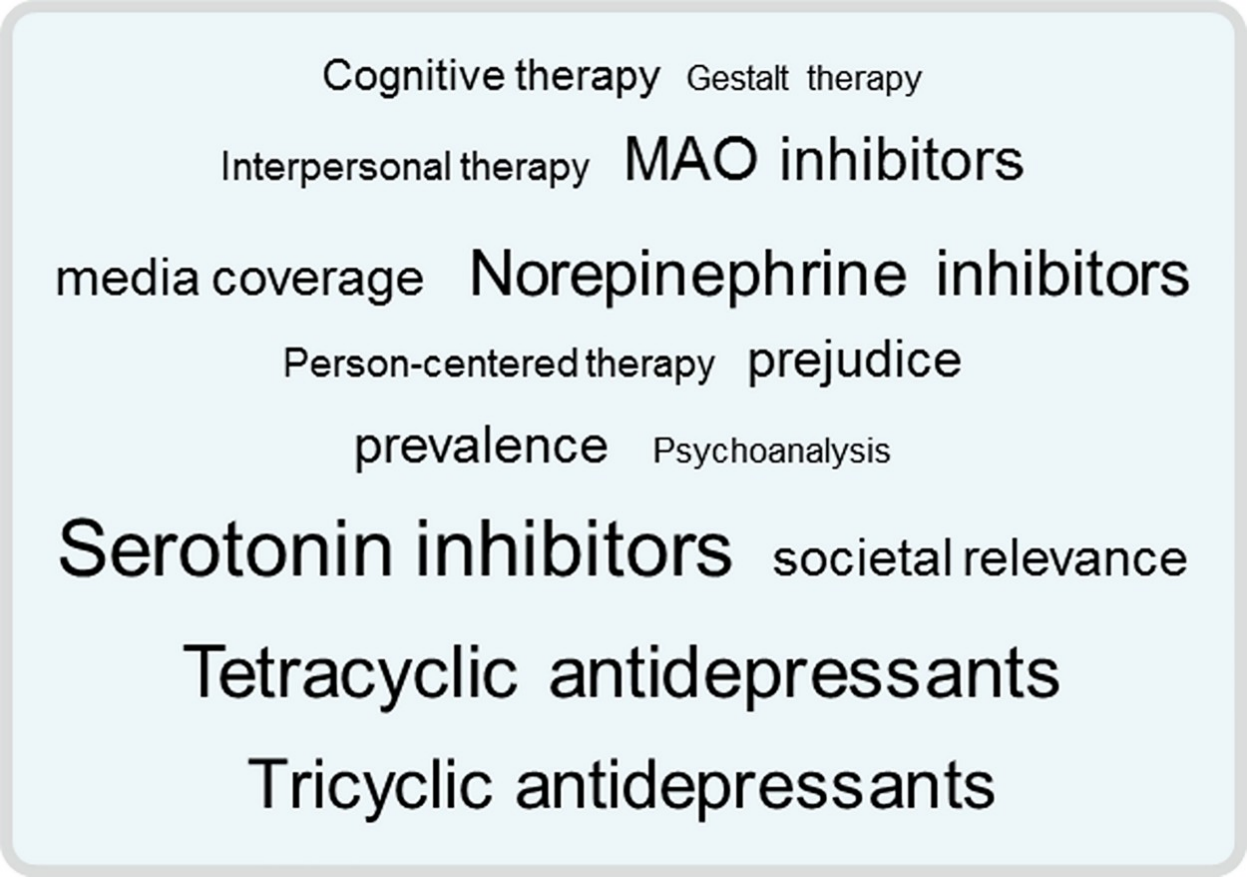
Tag cloud used in this study.

When users navigate between patches via tags (Fig 1), they need to estimate which navigation path will lead them to a patch with valuable information, or in other words, searchers follow the information scent. Information scent in social tagging systems depends on a user’s individual semantic associations as well as on the tag popularity of the tagging community (collective associations; [16,33,34]). Individual associations are activated in the searcher’s memory and will guide her or him to select tags that match with her or his associations. Collective associations in the form of popular tags will guide the searcher to select more popular tags which are displayed with a larger font size. Individual and collective associations are the fundamental building blocks of the extended information scent model [16], and literature on tagging presents individual and collective associations as the main factors that determine navigation between patches in social tagging systems [16,33,34].

Extending these findings, we raise the question as to whether, besides individual semantic associations, prior attitudes and thus confirmation bias also influence between-patch navigation in social tagging systems. A preliminary study showed that prior attitudes guided between-patch navigation (tag selection) as well as subsequent within-patch navigation (selection of blog posts on separate sites) [10]. In terms of information foraging, this result suggests that information scent depends on prior attitudes. So, regarding between-patch navigation, tags that were in line with prior attitudes were selected more often. Moreover, within-patch navigation, or the selection of tag-related blog posts, was related to prior attitudes. As an extension of these preliminary findings, we test in this study if the influence of prior attitudes on navigation can be enhanced or weakened by attitude confidence. We also test whether high credibility of the community circumvents the influence of the participants’ prior attitudes and increases their selection rate between and within patches independently of prior attitudes.

We align our expectations with existing literature on social tagging systems, where individual and collective factors independently influenced navigation and processing of information [10,16,33]. We assume that the searchers’ expected gain of desired information and therefore the information scent would depend on prior attitudes. Expected gain would be high when information is attitude consistent, and searchers would switch to more attitude-consistent patches by selecting respective tags. For blog posts, higher gain would be experienced from reading blog posts within attitude-consistent patches, and subsequent evaluation of blog posts should be more favourable when posts are attitude consistent. These effects should be stronger when attitude confidence is high compared to low. When source credibility is high, the expected gain should be higher for attitude-consistent as well as attitude- inconsistent patches, so overall, searchers should navigate more between and within patches.

### This study’s domain

To structure an investigation of a health-related information search scenario using the treatment of depression disorders, we made use of participants’ prior attitudes. In many countries, psychotherapy is thought to be more effective than antidepressants [35,36], although recent meta-analyses show equal efficacy, on a moderate level, for both treatments [37,38]. In Germany, for example, we previously found that antidepressants were considered to be moderately effective, whereas psychotherapy was even considered to be moderately to highly effective [10]. However, it should be noted that this issue is probably not highly controversial, as a combination of both therapies was recommended by a significant proportion of participants in our previous study [10].

To measure prior attitudes regarding the domain, and as part of the attitude confidence manipulation, we asked participants to provide arguments for and against psychotherapy and antidepressants. We propose that when a participant offers more arguments in favor of therapy and fewer against therapy, the overall evaluation is then positive, reflecting a positive attitude (in line with [39]). So, to measure prior attitudes, we built an index with the sum of pro and contra arguments for psychotherapy and antidepressants. This should ensure that treatment relevant attitudes are measured in a broad and personal sense, without topical constraints.

Finally, for the health-related domain of treatment for depression, we assume that individuals are accuracy-motivated when searching for information. A recent review has highlighted two different motivations that fuel confirmation bias [3]. First, accuracy-motivated individuals seek to select objectively correct information. That is, when accuracy motivation is high, individuals are more open to attitude-inconsistent information. This is because accuracy-motivated searchers are more influenced by cues that indicate objective correctness or perceived validity of information [3]. In contrast to this, defense motivation leads to selecting information that confirms prior attitudes and thus defends one’s self concept. For this study, our hypotheses are in line with the accuracy motivation theory, as we expect that for health-related searches, obtaining accurate information is more important to the searcher than protecting one’s self-concept.

### Overview and hypotheses

We used tags in a social tag cloud where antidepressant tags were larger than tags that related to psychotherapy. Both kinds of treatment are discussed in the context of health. Since there is a preference for psychotherapy over antidepressants in the population [40,41], we aimed to counter this preference to increase the likelihood of equal amounts of clicking on both treatments [10]. Before browsing the tag cloud, however, participants indicated their prior attitudes by providing arguments and efficacy ratings for antidepressants and psychotherapy. We began manipulation of the participants’ attitude confidence by having them recall experiences where they either felt confident or unconfident about their thoughts (see [25]). Subsequently, we presented a tag cloud which came from one of two communities that differed in alleged expertise in the domain. Via tag clouds, participants navigated among patches, and they were able to select multiple blog posts within each patch. All blog posts highlighted the efficacy of either psychotherapy or antidepressants in a positive way. We did not present any blog posts that compared both types of therapy, or any blog posts that presented studies with negative findings. To measure the searchers’ content evaluation as a consequence of navigation, we had them rate treatment efficacy again, after navigation. Finally, we conducted a recognition test for the blog posts participants had read.

We expected the following:

First, we expected our replication of the confidence manipulation [25] to be successful (H1a). When attitude confidence is high, people will select more attitude-consistent tags (H1b) and blog posts (H1c), compared to when attitude confidence is lower. That is, to the degree that people favour or disfavour treatments, confidence should moderate the influence of prior attitudes on attitude-consistent selection of tags and blog posts. Content evaluation in terms of treatment efficacy ratings after navigation will change accordingly (H1d).

Second, participants will recognize credibility of the tagging community (H2a). If a highly credible community (compared to a less credible community) provides content, tags (H2b) and blog posts (H2c) of this community should be selected more often. In the same vein, content gathered by a highly credible community will increase change of treatment efficacy ratings more compared to a less credible community (H2d).

A possible interaction effect between attitude confidence and source credibility remains an open question, since to the best of our knowledge, there are no background studies that have manipulated attitude confidence in combination with source credibility in online or offline information selection tasks. A second open question to be explored is whether knowledge acquisition will also be affected.

## Method

### Recruitment and participants

Participants were contacted via a mailing list. As an incentive, participants were offered the opportunity to take part in a lottery with 50 Euro Amazon gift certificates. Ethical approval was provided by the Ethical Committee of the Knowledge Media Research Center (LEK 2014/006). 138 participants out of a total of 331 persons who accessed the survey finished it. Five participants retracted their data. We dropped participants from the analysis who completed the study twice (n = 2), who did not provide prior attitudes(n = 2) or did not click on tags (n = 2). We also excluded outliers who scored in the attitude confidence manipulation check with a median absolute deviation greater than three (n = 2; [42]). 125 participants were included in the analysis. In the final sample, the age ranged from 19 to 64 years old (M = 24.74, SD = 6.27), and 90 participants were female (72%).

### Materials

**Fig 2:**
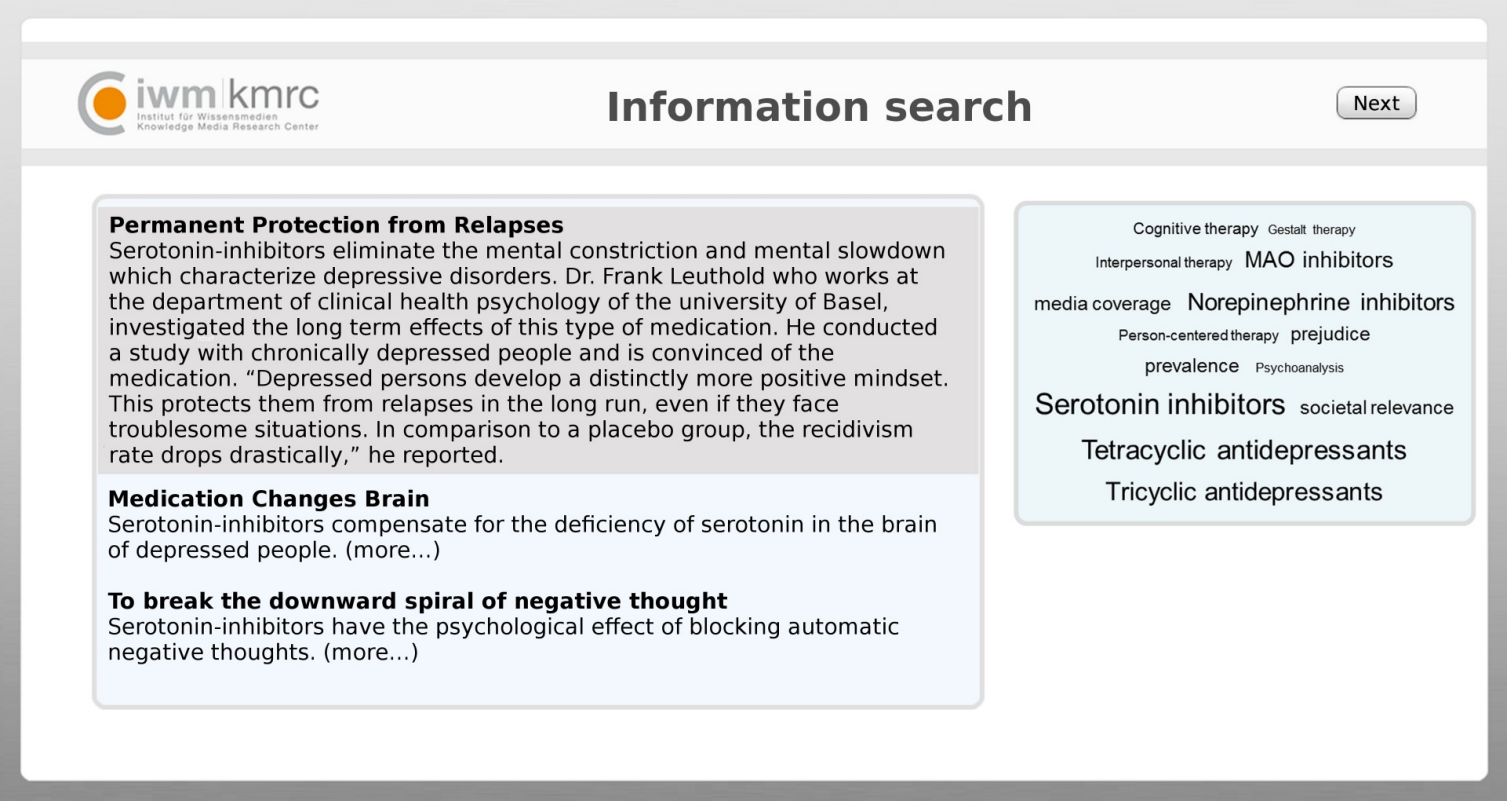
Social tagging environment used in the study. First published in the Journal of Medical Internet Research by [10].

For browsing through treatments, we provided a tagging environment that comprised two main sections (see Fig 2). The right part of the screen displayed 14 tags. Five tags represented psychotherapy and five tags represented antidepressants. Four tags were not relevant with respect to treatment (prejudice, media coverage, societal relevance, prevalence). Antidepressant tags were larger than psychotherapy tags, as can be seen in Fig 1 and Fig 2.

On the left part of the screen, blog posts were presented for each tag (Fig 2). Three blog posts were related to each tag. The content of the blog posts for antidepressants (15 posts) and psychotherapy (15 posts) was held constant with respect to number of arguments and length (mean 76.8 words, SD 6.1). Each post described a common symptom of depression disorders and scientific studies on the efficacy of the respective treatment. In a pilot study, we had made certain that the blog posts were equal in readability and credibility, and that there was no difference in the persuasiveness or quality of all of the arguments within the pairs of blog posts about antidepressants and psychotherapy (S1 Table). Initially, only the headline and the first sentence of each blog post were presented. In order to read the full blog post, participants had to click on the first sentence to expand the blog post.

### Design and procedure

**Fig 3:**
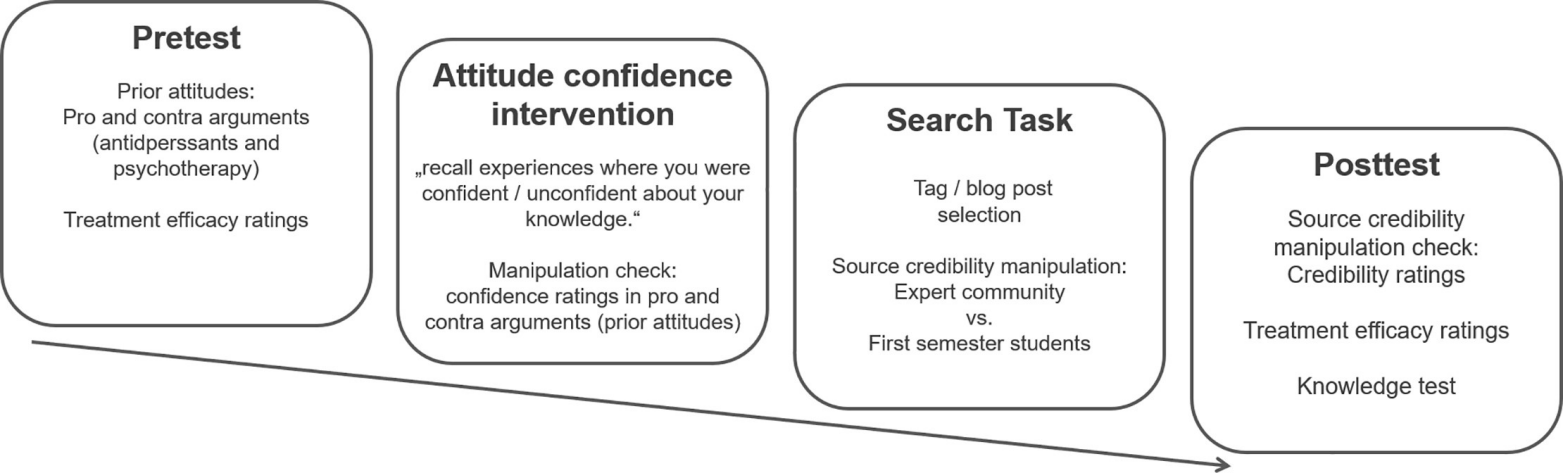
Experimental procedure.

The study comprised a 2 (attitude confidence: high, low) x 2 (source credibility: high, low) between-subjects design. Participants were randomly assigned to one of the four experimental conditions. On the first pages of the online survey, we welcomed participants, provided a brief review of the procedure of the study, asked participants to provide consent by clicking on the “continue” button, and requested basic demographic data. Then we asked them to state pro and contra arguments regarding antidepressants and psychotherapy (see box pretest in Fig 3). We also asked them to rate the efficacy of antidepressants and psychotherapy. After this, we manipulated *attitude confidence*. For an alleged unrelated study, we asked them to recall situations in which they had felt either confident or unconfident about their own thoughts [25]. After they had recalled such situations, we asked participants to think back to their arguments for and against psychotherapy and antidepressants. They rated how confident they felt about the arguments they had provided at the beginning of the study. This rating served as a manipulation check for attitude confidence.

In the search task, participants browsed a tag cloud that visualized treatments for depression. Tags related to antidepressants were larger than tags related to psychotherapy (Fig 2). We asked participants to search for information about the efficacy of treatments for depression, to provide advice for a hypothetical friend. The treatments were either psychotherapy or antidepressants. The tagging environment appeared for at least five minutes, after which participants could freely decide to browse further or to stop browsing tags and tag-related blog posts. After the navigation task, participants rated source credibility, which provided a manipulation check. Again, participants were asked to rate the efficacy of antidepressants and psychotherapy. Finally, participants filled out a retention test on the blog posts they had read. At the end, participants were debriefed and given the opportunity to leave comments on the study.

### Independent variables

#### Prior attitudes

As a measure of prior attitudes, we built an index of the sum of *pro* (positive value) and *contra* (negative value) *arguments* separately for psychotherapy and antidepressants. Each argument was counted by one rater, who coded each proposition for and against both treatments. (for examples, against antidepressants: “antidepressants are addictive”; for psychotherapy: “it helps when someone listens to your problems”). We validated the arguments against the treatment efficacy rating scale prior to navigation and found a correlation between the arguments and efficacy ratings for antidepressants (r = .18, p < .05), but not for psychotherapy (r = .06, ns.).

#### Attitude confidence

To manipulate attitude confidence, we adapted the experimental procedure used by Petty and colleagues (2002) to our study. After participants had provided their arguments for and against psychotherapy and antidepressants, we asked participants to recall situations in the past where they had felt either confident or unconfident about their own thoughts. The task was presented for a minimum of 5 minutes in which participants were asked to enter the situations in 5 input boxes.

#### Source credibility

**Fig 4:**
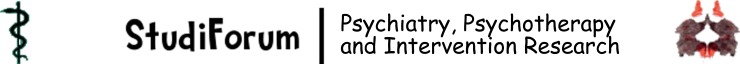
Translated version of the banner for the low source credibility group.

**Fig 5:**
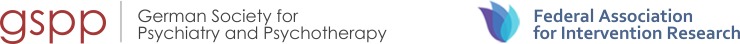
Translated version of the banner for the high source credibility group.

We presented banners which implied that either college students (low expertise) or domain experts (high expertise) had collected and tagged resources. At the top of the page in the tagging environment (above the visible space in Fig 2), either a banner of an online student forum (Fig 4), or a banner of alleged federal expert association (Fig 5) was displayed.

### Dependent variables

#### Selection of tags and blog posts

As indicators of participants’ search behavior, we assessed their clicks on tags and blog posts. The use of both measures as dependent variables allowed for an analysis of participants’ search behavior at various levels of elaboration: Whereas clicks on tags might indicate a general interest in the sources linked to this tag, they do not allow for any elaborate reception (i.e. reading) of related content. Therefore, we also analyzed the frequency of clicks on blog posts, which indicated participants’ interest in the posts’ specific content. We only counted clicks on those blog posts which were displayed for at least five seconds, which suggested that participants had spent more time reading those posts more thoroughly.

#### Treatment efficacy ratings

We calculated a score with subjective treatment efficacy ratings for antidepressants and psychotherapy. Participants rated the degree to which they agreed with the statements about the efficacy of both treatments, on a scale ranging from 1 (completely disagree) to 7 (completely agree). As an example: “There is scientific evidence that clearly demonstrates the efficacy of psychotherapy/antidepressants.” Efficacy ratings were assessed prior to navigation (see S2 Table; antidepressants Cronbach’s α = .79, psychotherapy Cronbach’s α = .88), and after navigation (antidepressants Cronbach’s α = .88, psychotherapy Cronbach’s α = .91).

#### Knowledge acquisition score

After navigation, participants filled out a multiple-choice test (1 target, 2 foils for each browsed blog post). For targets and foils, participants responded on a scale ranging from 1 (“completely wrong”) to 5 (“completely correct”). For example, the test questions for the blog post in Fig 2 is as follows: Target: “People who are treated with serotonin inhibitors show lowered risk of relapse in comparison to a control group.” Foil: “Serotonin inhibitors show a high tolerability.” We only analysed targets and foils that were related to a blog post that participants had clicked on. We recoded hits as 1 point and deleted items unrelated to blog posts that had been read, whereas the middle category (3: “I don’t know”) was coded as zero points. We calculated the total score separately for psychotherapy and antidepressants.

## Results

All of the following analyses were conducted with the statistical software R (v 3.5.0; [43]), including the packages “glmmADMB” (v 0.8.3.3; [44]), “tidyverse” (v 1.2.1; [45]). A full reproducible analysis script and data are available in the supplementary materials (see S1 Statistical Analyses).

### Prior attitudes

As expected [40,41], prior to navigation, participants evaluated psychotherapy more positively than antidepressants on the treatment efficacy rating scales (antidepressants: M = 4.23, SD = 0.93; psychotherapy: M = 5.69, SD = 0.76; t_124_ = 17.02, p < .001, d = 1.73). The same, but somewhat weaker tendency towards psychotherapy could be observed when analyzing the number of arguments regarding antidepressants (pro: M = 2.06, SD = 1.21; contra: M = 2.69, SD = 1.36) and psychotherapy arguments (pro: M = 2.91, SD = 1.39; contra: M = 1.92, SD = 1.32). These results showed a moderate tendency in favor of psychotherapy compared to antidepressants (pro arguments: t_124_ = 6.70, p < .001, d = 0.65; contra arguments: t_124_ = 6.12, p < .001, d = 0.57).

We calculated an index for prior attitudes by subtracting arguments in favor of each treatment from arguments against the respective treatment, so a negative prior attitudes score reflected a negative evaluation for the respective treatment, and a positive score a positive evaluation.

**Fig 6:**
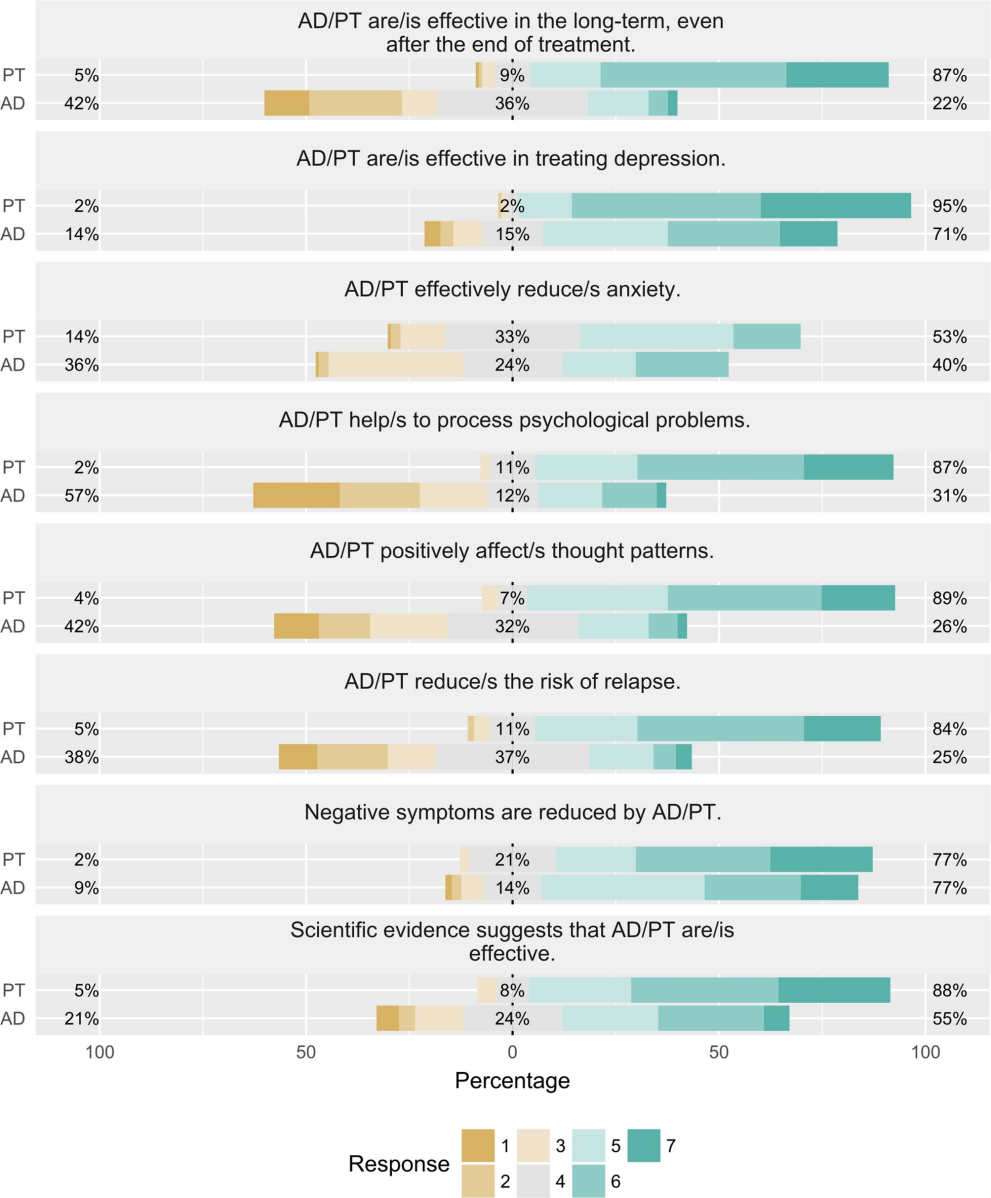
Treatment efficacy ratings for each item before navigation.

Treatment efficacy ratings were measured by the comparison of pretest ratings (before navigation) and posttest ratings (after navigation). Fig 6 and S2 Table provide an overview of the treatment efficacy rating items and response distribution.

### Manipulation checks

#### Attitude confidence

After participants recalled situations in which they had felt either confident or unconfident, they rated confidence in their own arguments for or against a depression treatment on a scale ranging from 1 (not at all) to 7 (highly). They also rated the degree to which the following qualities described their arguments: obvious, dubious, justified, credible, factual, well-founded, persuasive and objective (Cronbach’s α = .86). Those participants who during the manipulation had recalled situations in which they had felt confident in their own thoughts were also confident about their arguments about treatment (M = 5.10, SD = 0.75). In contrast, participants were less confident in their arguments after having recalled situations in which they had been unconfident (M = 4.81, SD = 0.74; t_123_ = 2.16, p = .03, d = 0.39). Our attitude confidence manipulation was thus shown to be effective and hypothesis H1a was supported.

#### Source credibility

After navigation, participants rated the credibility of the source of information on a scale ranging from 1 (not at all) to 7 (highly). They rated the degree to which the following qualities described the group who had compiled the blog posts: informed, conscientious, trustworthy, credible, and competent (Cronbach’s α = .89). Participants rated the source as more credible when the group consisted of alleged experts (M = 5.23, SD = 0.95), compared to students in their first semester who were rated as less credible (M = 4.88, SD = 0.87; t123 = 2.19, p = .04, d = 0.25). Thus, participants were able to recognize high and low expertise of the tagging community, and hypothesis H2a was supported.

### Navigation analyses

We analyzed navigation behavior with generalized linear mixed models [46], using the “glmmADMB” [44] package, to model the dependent variables counts of clicks on tags [H1b], and blog posts [H1c]). We first compared the empirical versus theoretical quantiles of tag and blog post counts visually, which suggested that the count data followed the Poisson distribution, so we marked? the models with a Poisson distribution with a log link.

As fixed factors, we included the independent variables credibility and confidence (both 0 = low, 1 = high) and the prior attitudes index. For the model predicting blog post counts, we included the number of clicks on tags as a covariate. Continuous predictors were standardized. To account for the within-subjects measure treatment [47], we included random by-participant and by-treatment intercepts.

For both models, visual inspection of the residual plots did not reveal any obvious deviations from homoscedasticity or normality. The model comparison strategy was to include all main (fixed) effects and random intercepts, and to compare this to the model including only the fixed intercept and the random intercepts (Tables 1 and 2, Step 1). Next, we included the hypothesized interaction term between attitude confidence and prior attitudes and compared this to the main effect model from Step 1 (Tables 1 and 2, Step 2). For exploratory purposes, we separately tested whether the model fit would further be improved by including the other two-way interaction terms (confidence x credibility, prior attitudes x credibility), and the three-way interaction term (prior attitudes x confidence x credibility). Significance of effects was obtained by means of a likelihood ratio test comparing the full model with the effect in question to the model without the effect in question [46].

**Table 1:** Tag selection.

**Step 1**		**b**	**SE**	**p**
	**Intercept**	.95	.10	< .001
	**Prior attitudes**	.03	.04	.52
	**Attitude confidence**	-.02	.10	.83
	**Source credibility**	.24	.10	.02
	(χ^2^(3) = 5.48, p = .14, R^2^ = .03)			
**Step 2**				
	**Prior attitudes X attitude confidence**	.01	.08	.87
	(χ^2^(1) = 0.25, p = .87, ΔR^2^ = .00)			
**Step 3**				
	**Attitude confidence X source credibility**	.14	.20	.51
	(χ^2^(1) = 0.44, p = .51, ΔR^2^ = .03)			
**Step 4**				
	**Prior attitudes X source credibility**	-.21	.08	.01
	(χ^2^(1) = 6.47, p = .01, ΔR^2^ = .00)			
**Step 5**				
	**Prior attitudes X attitude confidence X source credibility**	.02	.17	.88
	(χ^2^(1) = 0.02, p = .88, ΔR^2^ = .03)			

b, Beta coefficients with standardized continuous predictors; SE, Standard Error; R^2^, Nagelkerke R^2^ for fixed effects only.

**Table 2:** Blog post selection.

**Step 1**		**b**	**SE**	**p**
	**Intercept**	.29	.14	.04
	**Tags selected**	.53	.05	< .001
	**Prior attitudes**	.05	.06	.36
	**Attitude confidence**	.05	.13	.71
	**Source credibility**	.15	.13	.25
	(χ^2^(4) = 128.24, p = < .001, R^2^ = .33)			
**Step 2**				
	**Prior attitudes X attitude confidence**	.17	.08	.03
	(χ^2^(1) = 4.57, p = .03, ΔR^2^ = 0.01)			
**Step 3**				
	**Attitude confidence X source credibility**	-.47	.25	.07
	(χ^2^(1) = 3.41, p = .407, ΔR^2^ = .02)			
**Step 4**				
	**Prior attitudes X source credibility**	-.05	.08	.54
	(χ^2^(1) = 0.37, p = .53, ΔR^2^ = .00)			
**Step 5**				
	**Prior attitudes X attitude confidence X source credibility**	.07	.17	.68
	(χ^2^(1) = 0.17, p = .68, ΔR^2^ = .02)			

b, Beta coefficients with standardized continuous predictors; SE, Standard Error; R^2^, Nagelkerke R^2^ for fixed effects only.

#### Attitude confidence and navigation

**Tag selection:** We hypothesized that when attitude confidence is high (vs. low), people should select more attitude-consistent tags (H1b), depending on their prior attitudes. Results showed that there was no relationship between confidence and tag selection when including only main effects. It was shown that this model did not fit the data any better than the intercept-only model (Table 1, Step 1), and including the interaction term between confidence and prior attitudes did not improve the model fit either (Table 1, Step 2). Additionally, we checked for possible interaction effects among all predictors. Including the 2-way interaction between confidence and credibility (Table 1, Step 3) and the 3-way interaction (Table 1, Step 5) did not improve the model fit compared to step 1, whereas the model fit with main effects and the interaction between prior attitudes and confidence did improve mdel fit compared to step 1 (Table 1, Step 2). However, including the 2-way interaction between prior attitudes and source credibility did show a significant improvement of model fit compared to Step 2 (Table 1, Step 4), which will be discussed in the section below (Source credibility and navigation). Therefore, the hypothesis that attitude confidence would moderate the influence of prior attitudes on tag selection was not supported (H1b).

**Blog post selection:** We hypothesized that when attitude confidence is high (vs. low), people should select more attitude consistent blog posts (H1c), depending on their prior attitudes. For blog post selection (H1c, H2c), we used the same procedure for model comparison and the same independent variables as in the analysis of tag selection but additionally included the number of selected tags as a covariate. As Table 2 shows, tag selection predicted blog post selection (Step 1), and we found the hypothesized interaction between prior attitudes and attitude confidence (Step 2).

**Fig 7:**
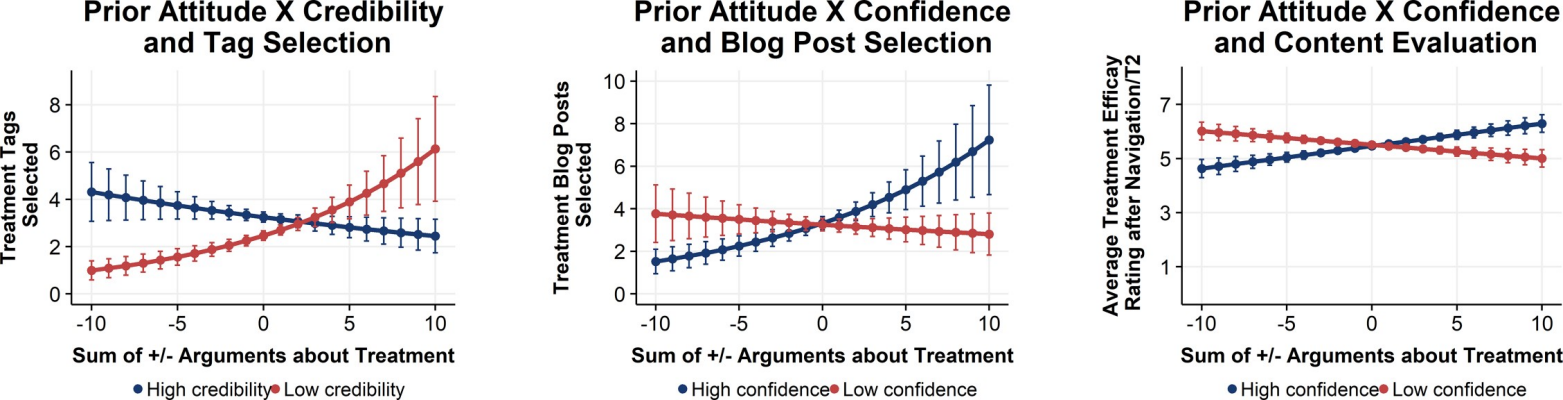
Overview of interaction effects.

To disentangle the interaction between prior attitudes an attitude confidence we did a subgroup analysis separately for the high (b = .13, SE = .06, p = .04) and low (b = -.02, SE = .05, p = .69) confidence groups, and plotted the predicted number of selected tags depending on prior attitudes (see Fig 7, middle panel). This finding supported our expectations that prior attitudes are only associated with the selection of blog posts when confidence is high (H1c).

#### Source credibility and navigation

**Tag selection:** We expected that participants would select more tags when source credibility was high, independent of their prior attitudes (H2b). As described above, the main effect model including the effect of source credibility did not fit the data. However, the model including the interaction between prior attitudes and source credibility did fit. To disentangle this interaction effect, we did a subgroup analysis for the high (b = -.05, SE = .05, p = .32) and low credibility (b = .16, SE = .06, p = .02 groups (see Fig 7, left panel), and plotted the predicted number of selected tags against prior attitudes. The result shows that only only the low credibility group selected tags which indicated attitude consistency with prior attitudes.

**Blog post selection:** We also expected that if the source credibility of the tagging community is high (vs. low), blog posts would be selected more often, independently of prior attitudes (H2c). Controlling for tag selection, however, we did not find any effect of source credibility on blog post selection (Table 2).

### Content evaluation analyses

To investigate evaluation of content, we ran linear mixed models [46], using the “lme4” [48] package, with the standardized, dependent variable treatment efficacy ratings after navigation. We first compared the empirical versus theoretical quantiles of tag and blog post counts visually, which suggested that the data followed a normal distribution.

As fixed factors, we included the independent variables source credibility and attitude confidence (both 0 = low, 1 = high) and the prior attitudes index. As covariates we included the treatment efficacy ratings before navigation and the number of clicks on blog posts. To control for the within-subjects factor treatment type, we included random intercepts for each participant and both treatment types. The continuous predictors tags and prior attitudes as well as the dependent variable treatment efficacy ratings were standardized [49].

For both models, visual inspection of the residual plots did not reveal any obvious deviations from homoscedasticity or normality. The model comparison strategy was to first include all main (fixed) effects and random intercepts (Table 3, Step 1), and to compare this to the model including only the fixed intercept and the random intercepts. Next, we included the fixed, hypothesized interaction term between attitude confidence and prior attitudes, and compared this to the main effect model from the first phase (Table 3, Step 2). For exploratory purposes, we separately tested whether the model fit would further be improved by including the other two-way, fixed interaction terms (confidence x credibility, prior attitudes x credibility), and the three-way, fixed interaction term (prior attitudes x confidence x credibility). Significance of effects was obtained by means of a likelihood ratio test comparing the full model with the effect in question to the model without the effect in question [46].

**Table 3:** Treatment efficacy ratings after navigation.

**Step 1**		**b**	**SE**	**p**
	**Intercept**	.03	.07	
	**Blog posts selected**	.22	.04	< .001
	**Treatment efficacy ratings before navigation**	.72	.04	< .001
	**Prior attitudes**	.02	.04	.57
	**Attitude confidence**	-.02	.09	.78
	**Source credibility**	-.03	.09	.72
	(χ^2^(4) = 190.92, p < .001, R^2^ = .64)			
**Step 2**				
	**Prior attitudes X attitude confidence**	.22	.07	.002
	(χ^2^(1) = 9.45, p = .002, ΔR^2^ = .01)			
**Step 3**				
	**Attitude confidence X source credibility**	.12	.17	.47
	(χ^2^(1) = 0.52, p = .47, ΔR^2^ = .01)			
**Step 4**				
	**Prior attitudes X source credibility**	.04	.07	.57
	(χ^2^(1) = 0.32, p = .57, ΔR^2^ = .00)			
**Step 5**				
	**Prior attitudes X attitude confidence X source credibility**	-.01	.14	.93
	(χ^2^(1) < 0.01, p = .99, ΔR^2^ = .01)			

b, standardized Beta coefficients; SE, Standard Error; R^2^, fixed effects only.

#### Attitude confidence and content evaluation

**Treatment efficacy ratings:** With H1d we expected that participants would keep their prior attitudes only when attitude confidence was high. When attitude confidence is low, participants should become more open to content of attitude inconsistent blog posts. The model including the interaction between prior attitudes and confidence did fit the data (Table 3, step 2). To disentangle this interaction effect, we did a subgroup analysis for the high (b = .17, SE = .06, p < .01) and low (b = -.11, SE = .05, p = .05) confidence groups, and plotted the predicted treatment ratings against prior attitudes. In support of H1d, the results showed that after controlling for blog post selection, only with high confidence were prior attitudes and attitude after navigation associated (Fig 7, right panel).

#### Source credibility and content evaluation

We expected that a high (vs. lower) source credibility would increase change of treatment efficacy ratings for attitude-consistent as well as attitude-inconsistent information (H2d). As shown in Table 3, there was no effect of source credibility on content evaluation measured by treatment efficacy ratings after navigation.

### Knowledge acquisition analyses

Additionally, we explored knowledge acquisition with linear regression. The goal was to evaluate whether knowledge acquisition would take place in a way consistent with prior attitudes. The criterion *correct responses* was entered as a dependent variable, and blog post selection, prior attitudes, attitude confidence, and source credibility were entered as predictors. As a random factor we included a by-participant intercept, whereas predictors were centered. The main effect model fit the data (χ^2^(4) = 98.99, p < .001). The number of selected antidepressant blog posts predicted the knowledge acquisition score (standardized b = .68, SE = .06, p < .001). However, the remaining predictors or including their interactions did not predict learning (all ps > .08). Our experimental manipulations of attitude confidence and source credibility were thus shown not to lead to more or less attitude-consistent knowledge acquisition.

### Overview and path analysis

**Fig 8:**
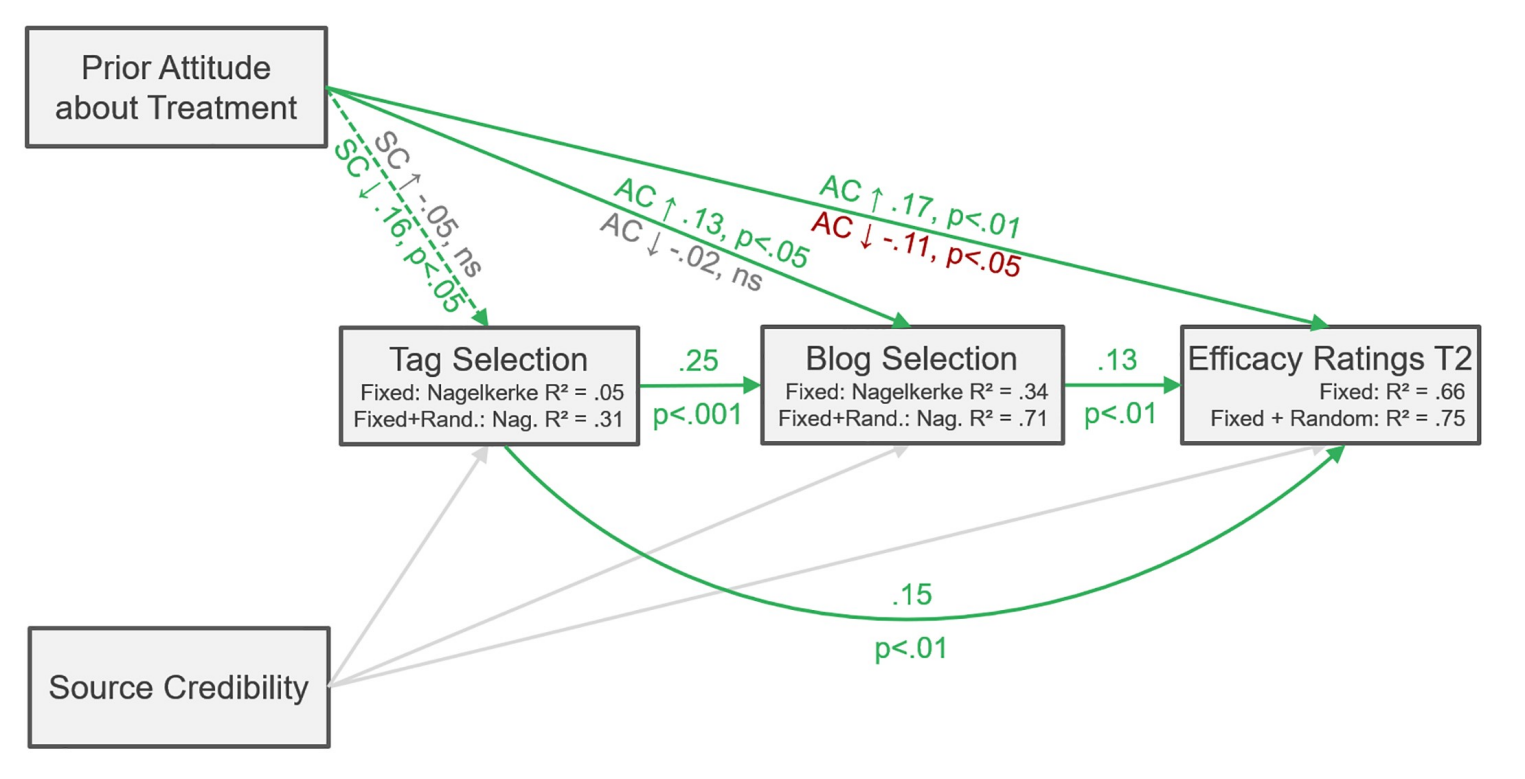
Path analysis. Note. AC = Attitude Confidence, SC = Source Credibility; Solid arrows = hypothesized effect; Dashed arrows = not hypothesized effect; Grey Arrows = not significant effects. Effects of the covariate treatment efficacy ratings T1 on blog post selection (b = .11, p < .01) and treatment efficacy ratings T2 (b = .71, p < .001) not shown. Subgroup analyses were conducted to provide separate group parameters when there was a significant association with prior attitudes.

To provide an overview on how attitude confidence and source credibility affect different stages of the navigation process, we conducted a confirmatory path analysis [50], using the R software with the packages “lme4” (v 1.1–17; [48]), and “piecewiseSEM” (v 2.0.2; [51]). We used the piecewise structural equation modelling approach, as it allows for including models for Poisson distributed count data [50]. The approach makes it possible to evaluate multiple causal hypotheses simultaneously within a single network of connected nodes [51]. Therefore, we evaluated separate component models in the form of regression equations, as reported above (Table 1, Step 4; Table 2, Step 2; Table 3, Step 2). However, tests of directed separation showed that 2 paths of tag selection on treatment efficacy ratings T2, and treatment efficacy ratings T1 for blog post selection were missing, which were included in the final model. The final model did fit the data, and no significant paths were omitted (C = 1.50, P = .83; [52]; see Fig 8).

## Discussion

In this study, our aim was to investigate how social tag clouds would influence a pre-existing confirmation bias. Searchers would be expected to be more open to social tags and blog posts that challenged their prior attitudes if they had little confidence in their prior attitudes. We also aimed to test whether searchers would correctly evaluate source credibility, and if in turn they would explore more content and be more open to content when they perceived the credibility of the tagging community to be high. We found that searchers exhibited more favourable attitudes towards psychotherapy compared to antidepressants and that searchers correctly recognized source credibility in terms of high and low community expertise. Results also showed that our replication of the attitude-confidence manipulation was successful, making it possible to rely on its effects. In the navigation process, source credibility had influence only on the early stage of tag selection, whereas confidence had consequences later, when participants were selecting blog posts and subsequently evaluating content.

We acknowledged that people tend to confirm their prior attitudes. However, specifically when prior attitudes were pronounced, and attitude confidence was high (vs. low), confirmation bias increased in blog post selection, but not in tag selection. Moreover, when attitude confidence was high, prior attitudes were positively associated with evaluation of attitude-consistent content. When attitude confidence was low, the effect was even reversed, that is, attitude-inconsistent content was evaluated more favourably.

We also found that when credibility was high, the influence of prior attitudes on tag and blog post selection was eliminated. By contrast, when source credibility was low, prior attitudes guided selection of tags and blog posts, showing positive association with the selection of attitude-consistent tags.

### Attitude confidence

In general, people tend to be overly confident about their own knowledge [13,17,18], but particularly in the context of health-related information search, confidence in one’s prior attitudes may vary. Having chosen a health-related domain, we therefore used manipulation that had the potential to counter overconfidence. In this scenario, we expected that the metacognitive aspect of attitude confidence, that is, confidence in the validity of one’s own arguments, would have consequences for the search and cognitive processing of health-related information.

We had expected that the influence of prior attitudes would depend on confidence in information search and content evaluation. When they possessed high confidence, searchers would be expected to select and favourably evaluate attitude-consistent information. We found this influence of confidence only for the evaluative stage of information search, or in terms of information foraging [15], the activity of within-patch exploitation. This included the selection of blog posts as well as the subsequent content evaluation. When confidence was high, participants showed a tendency to select and favourably evaluate attitude-consistent information, but when confidence was low, there was no influence of prior attitudes on navigation.

In the light of the self-validation hypothesis, from which the original attitude confidence manipulation was derived [25], the findings seem to support the assumption that searchers in the tagging environment were motivated primarily to look for accuracy. The self-validation hypothesis states that only thoughts that are perceived as valid determine attitudes and related processing of information. If confidence is high, one’s own thoughts or arguments are perceived as valid, and consequently one’s own arguments should have a high impact on navigation and content evaluation, in contrast to when confidence is low. In support of this theory, we found an influence of high attitude confidence on the evaluative stage of search, in which content was selected and evaluated. More surprisingly, but in line with this train of thought, when attitude confidence was low, the effect of prior attitudes on content evaluation was even reversed, and participants rated attitude-inconsistent content more favourably.

Finally, to the best of our knowledge, this is the first study that has directly replicated the metacognitive confidence manipulation [25]. It is therefore worthwhile to compare the effects of the manipulation in both studies. Interestingly, the original study showed a large effect of the manipulation. Our replication, however, showed a small to moderate effect on the same scale. Moreover, both the high and the low attitude confidence groups were above the midpoint of the scale. This finding is not surprising, considering that most people tend to be overly confident in a wide range of domains [13,19].

### Evaluation of source credibility

We had expected that source credibility would influence information search and the resulting content evaluation, independently of prior attitudes. Information search consisted of two consecutive processes: First, selection of topics (tags), and second, selection of in-depth, valenced information (blog posts). The topic-oriented tag selection illustrated the between-patch activity in terms of information foraging theory [15], where information foragers switch among information environments (patches). Source credibility only affected this exploratory, uncertain information selection process where the searcher needed to estimate which tag would lead to the most valuable information. We also observed an interplay of source credibility and prior attitudes. That is, prior attitudes had no influence on tag selection only when source credibility was high, and when source credibility was low, participants with increasingly positive attitudes towards a treatment selected that respective treatment tag more often.

In terms of a confirmation bias, the finding that under high source credibility prior attitudes were not associated with the selection of attitude consistent tags supports the accuracy motivation theory [3]. That is, participants valued objectively correct information, which they would more likely get from a highly credible source. In contrast to the accuracy motivation theory, the defense motivation theory would predict that prior attitudes would be negatively associated with the selection of attitude inconsistent tags when source credibility is high, since high credibility of attitude- inconsistent information would pose a threat. For the high source credibility condition, the finding is in line with our assumption that participants would be likely to be motivated by a desire for accuracy, due to the health-related context.

But for the low source credibility condition, searchers might have been acting less out of accuracy or defense motivation. If searchers were highly defense-motivated, participants would have avoided attitude-consistent information under low source credibility, as this potentially could have made them aware of their own questionable position. If searchers were highly accuracy-motivated, prior attitudes should not have affected tag selection, as these searchers would want to aim for high quality information [3]. A possible explanation could be that if searchers in the low credibility source condition were low in both accuracy as well as defense motivation, they might simply have been guided by their confirmation bias.

Finally, participants successfully identified tagging communities as more credible when high (vs. low) community expertise was indicated by banners. This is an interesting finding, since for platforms offering user-generated content such as blog posts or forums, users now generally evaluate content as more credible if other users with similar demographics (not experts) generated content [14]. This shows the potential of highlighting the expertise and credibility of the community for the searcher on social tagging platforms.

### Limitations and future work

In this study, tags were related to a set of different blog posts, so at first sight, tags were ambiguous with respect to related content. If one encountered the tag “antidepressant”, it was not clear what the related documents were about, or if these documents supported or refuted claims about the efficacy of antidepressants. In fact, purely semantic processing of tags was forced by the selection of the tags in our tagging environment. So when prior attitudes were in line with tag selection in this study, it implies that people might have been showing that the testing strategy documented in confirmation bias literature is a good one to use [11]. But tags that are provided in real tagging systems are also evaluative, motivational and social in nature [53,54]. We think it would be desirable to pursue studies that investigate the evaluative nature of processing of tags, in order to include evaluative processes in tagging theories as well (e.g. [55]).

Moreover, issue involvement has been shown to be an important moderator with respect to attitude-consistent information processing [3]. In this study, we did not measure involvement, which would be interesting for future studies.

As already mentioned, all of the blog posts people had access to were positive about the efficacy of either psychotherapy or antidepressants as treatments for depression. There were no blog posts that refuted the efficacy of either type of therapy. Future studies should include also neutral and negative statements about efficacy.

### Outlook

We found that tag clouds may offer a way to counter confirmation bias in online health-related tagging environments. However, the extent of confirmation bias also depends on individual cognitive processes, such as confidence in one's attitudes, and on the credibility of the community providing the information. Manipulating attitude confidence offers an effective and uncomplicated intervention to reduce bias among individuals. Highlighting the credibility of a source helps to increase the impact of health-related online information and also reduce bias. With respect to confirmation bias, there is a concern that online aggregation mechanisms act as echo chambers, reinforcing people's attitudes, trapping them in filter bubbles (e.g. [26]). Although some expect the effect of the bubbles to be large [26], others expect the effect to be small [56]. With the findings of the study presented here, however, we hope to contribute to a more nuanced discussion about this topic.

## Supporting information

S1 Coding Details Describing variables in rawdata. (CSV)

S1 Rawdata Rawdata analysed for the current study. (CSV)

S2 Rawdata Data from the pilot study. (CSV)

S1 Statistical Analyses Script of statistical analysis of rawdata conducted in R. (R)

S1 Table Blog post efficacy ratings in the pilot study. (DOCX)

S2 Table General treatment efficacy ratings of antidepressants and psychotherapy. (DOCX)
