# Supplementary material for: Attitude confidence and source credibility in information foraging with social tags
Source: PLoS One. 2019 Jan 15;14(1):e0210423. doi: 10.1371/journal.pone.0210423 (PMC6333359; doi:10.1371/journal.pone.0210423)
Supplement: S1 Table — (DOCX) [file pone.0210423.s005.docx]

**Table 1.** **Comparison of blog post efficacy ratings in the pilot study.**

|  |  | **“The blog post is convincing regarding treatment efficacy.”** | | | | **“The blog post is comprehensible”** | | | | **“The quality of the arguments in the blog post is very high.”** | | | |
| --- | --- | --- | --- | --- | --- | --- | --- | --- | --- | --- | --- | --- | --- |
| *Treatment* | *Blog Post Number* | *Mean* | *SD* | *t* | *p* | *Mean* | *SD* | *t* | *p* | *Mean* | *SD* | *t* | *p* |
| Behavrioural Therapy | 1 | 5.44 | 1.16 |  |  | 6.25 | 1.14 |  |  | 5.19 | 1.28 |  |  |
| MAO Inhibitors | 1 | 5.03 | 1.20 | 1.490 | .1463 | 6.31 | 0.93 | -0.32 | .7512 | 4.53 | 1.44 | 1.996 | .0548 |
|  |  |  |  |  |  |  |  |  |  |  |  |  |  |
| Interpersonal Therapy | 2 | 5.09 | 1.44 |  |  | 6.00 | 1.11 |  |  | 4.94 | 1.34 |  |  |
| Serotonin-Inhibitors | 1 | 5.00 | 1.44 | 0.303 | .7636 | 5.66 | 1.33 | 1.23 | .2272 | 4.41 | 1.46 | 1.503 | .1430 |
|  |  |  |  |  |  |  |  |  |  |  |  |  |  |
| Interpersonal Therapy | 1 | 4.97 | 1.28 |  |  | 6.28 | 0.85 |  |  | 4.66 | 1.60 |  |  |
| Tricyclic Antidepressants | 1 | 5.00 | 1.68 | -0.102 | .9194 | 6.25 | 1.08 | 0.17 | .8650 | 4.66 | 1.73 | 0.000 | .9990 |
|  |  |  |  |  |  |  |  |  |  |  |  |  |  |
| Person-Centered Therapy | 1 | 4.97 | 1.47 |  |  | 6.00 | 1.05 |  |  | 4.56 | 1.66 |  |  |
| Tetracyclic Antidepressants | 1 | 4.66 | 1.21 | 1.186 | .2446 | 6.00 | 1.19 | 0.00 | .9990 | 4.44 | 1.41 | 0.383 | .7041 |
|  |  |  |  |  |  |  |  |  |  |  |  |  |  |
| Person-Centered Therapy | 3 | 5.00 | 1.34 |  |  | 6.25 | 0.98 |  |  | 4.69 | 1.65 |  |  |
| Serotonin-Inhibitors | 2 | 5.50 | 1.27 | -2.033 | .0507 | 6.25 | 0.88 | 0.00 | .9990 | 5.16 | 1.32 | -1.507 | .1419 |
|  |  |  |  |  |  |  |  |  |  |  |  |  |  |
| Psychodynamic Therapy | 2 | 4.50 | 1.61 |  |  | 5.94 | 1.05 |  |  | 4.03 | 1.84 |  |  |
| Serotonin-Inhibitors | 3 | 4.72 | 1.17 | -0.960 | .3443 | 6.06 | 0.98 | -0.58 | .5637 | 4.22 | 1.74 | -0.692 | .4942 |
|  |  |  |  |  |  |  |  |  |  |  |  |  |  |
| Gestalt Therapy | 2 | 5.16 | 1.27 |  |  | 6.25 | 0.95 |  |  | 4.75 | 1.37 |  |  |
| Tetracyclic Antidepressants | 2 | 5.06 | 1.44 | 0.373 | .7118 | 6.25 | 1.08 | 0.00 | .9990 | 4.78 | 1.43 | -0.111 | .9125 |

|  |  | **“The blog post is convincing regarding treatment efficacy.”** | | | | **“The blog post is comprehensible”** | | | | **“The quality of the arguments in the blog post is very high.”** | | | |
| --- | --- | --- | --- | --- | --- | --- | --- | --- | --- | --- | --- | --- | --- |
| *Treatment* | *Blog Post Number* | *Mean* | *SD* | *t* | *p* | *Mean* | *SD* | *t* | *p* | *Mean* | *SD* | *t* | *p* |
| Gestalt Therapy | 3 | 5.19 | 1.31 |  |  | 6.19 | 0.97 |  |  | 4.88 | 1.41 |  |  |
| Tricyclic Antidepressants | 3 | 4.69 | 1.33 | 1.910 | .0655 | 6.06 | 0.95 | 0.60 | .5549 | 4.41 | 1.50 | 1.557 | .1297 |
|  |  |  |  |  |  |  |  |  |  |  |  |  |  |
| Behavrioural Therapy | 2 | 5.47 | 1.11 |  |  | 6.34 | 0.83 |  |  | 5.19 | 1.40 |  |  |
| Noradrenalin Inhibitors | 2 | 4.69 | 1.47 | 3.498 | .0014 | 6.12 | 1.26 | 1.05 | .3041 | 4.34 | 1.56 | 2.862 | .0075 |
|  |  |  |  |  |  |  |  |  |  |  |  |  |  |
| Behavrioural Therapy | 3 | 5.34 | 0.97 |  |  | 6.19 | 0.90 |  |  | 4.84 | 1.27 |  |  |
| Tetracyclic Antidepressants | 3 | 5.12 | 1.50 | 1.000 | .3251 | 5.97 | 1.18 | 1.27 | .2138 | 4.78 | 1.66 | 0.232 | .8179 |
|  |  |  |  |  |  |  |  |  |  |  |  |  |  |
| Psychodynamic Therapy | 3 | 4.16 | 1.32 |  |  | 4.81 | 1.38 |  |  | 3.84 | 1.57 |  |  |
| Noradrenalin Inhibitors | 3 | 4.66 | 1.29 | -1.856 | .0730 | 5.62 | 0.94 | -3.18 | .0034 | 4.19 | 1.60 | -1.098 | .2806 |
|  |  |  |  |  |  |  |  |  |  |  |  |  |  |
| Interpersonal Therapy | 3 | 5.09 | 1.63 |  |  | 6.00 | 0.98 |  |  | 4.22 | 1.77 |  |  |
| MAO Inhibitors | 2 | 4.97 | 1.66 | 0.453 | .6536 | 5.66 | 1.52 | 1.16 | .2552 | 4.25 | 1.63 | -0.106 | .9166 |
|  |  |  |  |  |  |  |  |  |  |  |  |  |  |
| Person-Centered Therapy | 2 | 5.12 | 1.39 |  |  | 6.06 | 1.22 |  |  | 4.66 | 1.56 |  |  |
| Tricyclic Antidepressants | 2 | 5.06 | 1.46 | 0.220 | .8271 | 6.16 | 1.05 | -0.41 | .6813 | 5.00 | 1.44 | -1.216 | .2330 |
|  |  |  |  |  |  |  |  |  |  |  |  |  |  |
| Gestalt Therapy | 1 | 4.50 | 1.48 |  |  | 6.19 | 1.00 |  |  | 4.03 | 1.53 |  |  |
| Noradrenalin Inhibitors | 1 | 5.25 | 1.39 | -2.823 | .0082 | 6.19 | 1.03 | 0.00 | .9990 | 4.78 | 1.56 | -3.000 | .0053 |
|  |  |  |  |  |  |  |  |  |  |  |  |  |  |
| MAO Inhibitors | 3 | 4.34 | 1.60 |  |  | 5.84 | 1.37 |  |  | 4.06 | 1.63 |  |  |
| Psychodynamic Therapy | 1 | 4.50 | 1.30 | -0.487 | .6299 | 6.09 | 0.96 | -1.03 | .3092 | 3.81 | 1.35 | 1.000 | .3251 |
| Note. N = 32 participants had been asked three questions for each blog post which were used as blog posts in the current study. 15 blog post pairs with similar main arguments were constructed and each pair was tested for difference in efficacy, readability and argument quality with paired t-Tests. Critical p value after Dunn-Šidák Correction for 45 pairwise comparisons is 0.0011. Participants agreed on a range from 1 (not at all) to 7 (completely agree). | | | | | | | | | | | | | |
