# Supplementary material for: Attitude confidence and source credibility in information foraging with social tags
Source: PLoS One. 2019 Jan 15;14(1):e0210423. doi: 10.1371/journal.pone.0210423 (PMC6333359; doi:10.1371/journal.pone.0210423)
Supplement: S2 Table — (DOCX) [file pone.0210423.s006.docx]

**Table 2. General treatment efficacy ratings of antidepressants and psychotherapy.**

|  | **Before Navigation** | |  | **After Navigation** | |
| --- | --- | --- | --- | --- | --- |
|  | *M* | *SD* |  | *M* | *SD* |
| Psychotherapy is effective in the long-term, even after the end of treatment. | 5.73 | 1.09 |  | 6.01 | 1.04 |
| Antidepressants are effective in the long-term, even after the end of treatment. | 3.45 | 1.49 |  | 4.43 | 1.75 |
|  |  |  |  |  |  |
| Psychotherapy is effective in treating depression. | 5.74 | 1.14 |  | 6.12 | 0.96 |
| Antidepressants are effective in treating depression. | 5.02 | 1.47 |  | 5.42 | 1.34 |
|  |  |  |  |  |  |
| Psychotherapy effectively reduces anxiety. | 5.58 | 0.99 |  | 5.66 | 0.93 |
| Antidepressants effectively reduce anxiety. | 4.57 | 1.5 |  | 5.21 | 1.43 |
|  |  |  |  |  |  |
| Psychotherapy helps to process psychological problems. | 6.11 | 0.92 |  | 6.22 | 0.96 |
| Antidepressants help to process psychological problems. | 3.31 | 1.81 |  | 4.38 | 1.83 |
|  |  |  |  |  |  |
| Psychotherapy positively affects thought patterns. | 5.68 | 1.01 |  | 6.21 | 1.01 |
| Antidepressants positively affect thought patterns. | 3.62 | 1.48 |  | 4.48 | 1.62 |
|  |  |  |  |  |  |
| Psychotherapy reduces the risk of relapse. | 5.54 | 1.13 |  | 5.88 | 1.05 |
| Antidepressants reduce the risk of relapse. | 3.64 | 1.5 |  | 4.53 | 1.51 |
|  |  |  |  |  |  |
| Negative symptoms are reduced by psychotherapy. | 5.52 | 1.02 |  | 5.81 | 1.11 |
| Negative symptoms are reduced by antidepressants. | 5.13 | 1.26 |  | 5.47 | 1.27 |
|  |  |  |  |  |  |
| Scientific evidence clearly demonstrates the efficacy of psychotherapy. | 5.57 | 1.14 |  | 6.02 | 1.00 |
| Scientific evidence clearly demonstrates the efficacy of antidepressants. | 5.23 | 1.23 |  | 5.84 | 1.21 |
